# Aging, Empathy, and Prosocial Behaviors During the COVID-19 Pandemic

**DOI:** 10.1093/geroni/igab046.2739

**Published:** 2021-12-17

**Authors:** Isu Cho, Ryan Daley, Tony Cunningham, Elizabeth Kensinger, Angela Gutchess

**Affiliations:** 1 Brandeis University, Waltham, Massachusetts, United States; 2 Boston College, Chestnut Hill, Massachusetts, United States

## Abstract

Previous literature has shown age-related increases in prosociality (i.e., the tendency to engage in behaviors that benefit others). Can such age-related differences be observed during the COVID-19 pandemic, or would young adults’ higher levels of COVID-19-related stress alter the relation between age and prosociality given the prior findings that stress may promote prosocial behaviors? Can empathy, one of the factors highly related to prosociality, explain any observed age-related differences? The current study examined the above questions, as well as whether age differences exist in target of prosocial behaviors (i.e., distant- versus close-others). To this end, participants (aged 18-89) enrolled in an ongoing study examining their experiences during the COVID-19 pandemic. They were asked to complete a series of surveys on dispositional empathy and prosocial behaviors during the pandemic. In the present analyses, the data were used from 330 participants from the USA who completed all of the surveys. Compared to younger adults, results indicate that older adults showed greater prosocial behaviors during the pandemic despite their higher risk of physical-health complications from COVID-19. Unexpectedly, empathy did not explain such age-related increases in prosocial behaviors even though it was positively related to individuals’ prosociality. Interestingly, older adults reported increased prosocial behaviors towards close-others (i.e., family, friends) compared to young adults, suggesting that older adults seem to devote more resources into emotionally meaningful relationships. The current study contributes to our understanding of how prosociality differs with age during the stressful period of need that marks the COVID-19 pandemic.

